# Molecular, Biochemical and Genetic Characteristics of BSE in Canada

**DOI:** 10.1371/journal.pone.0010638

**Published:** 2010-05-14

**Authors:** Sandor Dudas, Jianmin Yang, Catherine Graham, Markus Czub, Tim A. McAllister, Michael B. Coulthart, Stefanie Czub

**Affiliations:** 1 Canadian and OIE Reference Laboratories for BSE, Canadian Food Inspection Agency Lethbridge Laboratory, Lethbridge, Alberta, Canada; 2 Agriculture and Agri-Food Canada Research Centre, Lethbridge, Alberta, Canada; 3 College of Veterinary Medicine, China Agricultural University, Beijing, China; 4 Faculty of Veterinary Medicine, University of Calgary, Calgary, Alberta, Canada; 5 Canadian Creutzfeldt-Jakob Disease Surveillance System, Public Health Agency of Canada, Winnipeg, Manitoba, Canada; Institute of Evolutionary Biology (CSIC-UPF), Spain

## Abstract

The epidemiology and possibly the etiology of bovine spongiform encephalopathy (BSE) have recently been recognized to be heterogeneous. In particular, three types [classical (C) and two atypical (H, L)] have been identified, largely on the basis of characteristics of the proteinase K (PK)-resistant core of the misfolded prion protein associated with the disease (PrP^res^). The present study was conducted to characterize the 17 Canadian BSE cases which occurred prior to November 2009 based on the molecular and biochemical properties of their PrP^res^, including immunoreactivity, molecular weight, glycoform profile and relative PK sensitivity. Two cases exhibited molecular weight and glycoform profiles similar to those of previously reported atypical cases, one corresponding to H-type BSE (case 6) and the other to L-type BSE (case 11). All other cases were classified as C-type. PK digestion under mild and stringent conditions revealed a reduced protease resistance in both of these cases compared to the C-type cases. With Western immunoblotting, N-terminal-specific antibodies bound to PrP^res^ from case 6 but not to that from case 11 or C-type cases. C-terminal-specific antibodies revealed a shift in the glycoform profile and detected a fourth protein fragment in case 6, indicative of two PrP^res^ subpopulations in H-type BSE. No mutations suggesting a genetic etiology were found in any of the 17 animals by sequencing the full PrP-coding sequence in exon 3 of the *PRNP* gene. Thus, each of the three known BSE types have been confirmed in Canadian cattle and show molecular characteristics highly similar to those of classical and atypical BSE cases described from Europe, Japan and the USA. The occurrence of atypical cases of BSE in countries such as Canada with low BSE prevalence and transmission risk argues for the occurrence of sporadic forms of BSE worldwide.

## Introduction

Prion diseases are invariably fatal neurological diseases that usually cause severe spongiform change in the brain associated with an accumulation of a misfolded isoform of the prion protein (PrP^Sc^) [1]. This misfolded isoform is conformationally distinct from the cellular prion protein (PrP^C^) and exhibits a feature important for diagnostic purposes – the partial resistance to proteinase K (PK) digestion. The PK-resistant core of PrP^Sc^ is denoted as PrP^res^ [2]. PrP^res^ is often used for the detection of prion diseases, and its molecular features are useful to characterize the type of prion disease in individual cases. PrP^res^ displays both variation in molecular size of the residual protein core, based on variation in the location of PK cleavage sites, and micro heterogeneity based on differential occupancy of two N-linked glycosylation sites in PrP. This leads to di-, mono- and unglycosylated protein subpopulations (glycoforms) that can vary in relative abundance as assessed by their reactivities on Western immunoblots. Variation in PK cleavage also results in changes in immunoreactivity profile of PrP^res^, as key epitopes may be present or absent in the PK-resistant core. Different prion disease types may also vary in PrP^res^ conformational stability [3], [4].

Until recently, it was widely assumed that bovine spongiform encephalopathy (BSE) in cattle consisted of only a single, epidemiologically and biologically homogeneous type. This was based largely on the fact that experimental transmissions of the BSE agent to laboratory mice yielded a uniform lesion profile in the brain with invariable incubation time, irrespective of the source of BSE inoculum, but also on uniformity of PrP^res^ characteristics [5]–[8]. The lesion profiles and incubation times in these mice were also undistinguishable from those seen in mice inoculated with human variant Creutzfeldt-Jakob disease (vCJD). The PrP^res^ from these patients and animals also showed similar molecular weights and glycoform profiles, using Western blot (WB) analyses [9], [10]. These results strongly suggested that BSE was caused by a single strain of agent, and that exposure to the BSE agent was the most likely cause of human vCJD [11], [12].

However, in 2004, two new “atypical” types of BSE were identified in Italy and France. The Italian type was named bovine amyloidotic spongiform encephalopathy (BASE), because of the unusual and widespread occurrence of PrP^Sc^-containing amyloid plaques in brain tissue [13]. Molecular characterization of the PrP^res^ from these cases revealed a more equal ratio of immunoreactivities for di- and monoglycosylated glycoforms and a lower molecular weight of the unglycosylated glycoform than seen in previous BSE cases. Thus indicating a different PK-cleavage site and supporting the notion of a novel prion disease type. The lower molecular weight also prompted designation of such cases as “L-type” BSE, as distinct from classical or “C-type” BSE. The French type had a distinctly higher molecular weight of PrP^res^ bands compared to C-type BSE resulting in it being called high or “H-type” BSE [14]. At least one of these two types of BSE, together known as atypical BSE, have since been detected in Germany [3], Japan [15], the Netherlands [2], Poland [16], Sweden [2], Switzerland [17], the United Kingdom [18] and the United States [19]; all in cattle 8 years of age and older. Two as-yet-unclassified cases of atypical BSE have also been reported in Japan and Belgium [20], [21].

Experiments with atypical H- and L-type isolates have demonstrated their transmissibility to cattle as well as to mice expressing the bovine prion protein and wild-type mice. These isolates clearly differ from C-type BSE because they display unique incubation periods, PrP^res^ deposition patterns, and patterns of histological lesions [20], [22], [23]. While the C-type BSE cases that occurred as part of the outbreak in the U.K., Europe, Japan and Canada were believed to be caused by the consumption of contaminated feedstuffs [24], the origins of H- and L-type BSE are unknown. It has been speculated that atypical BSE may be sporadic or genetically caused, and indeed, strong evidence for a causative role of high-penetrance genetic mutations has been found in at least one case [25]. Either cause could be linked to the origin of C-type BSE but the question of origin may never be definitively answered [23]. The full risks presented to human health by atypical BSE remain unknown, but data is available suggesting that L-type BSE may be zoonotic [26], [27].

As of November 2009, 17 Canadian cases of BSE have been confirmed. In order to assess their potential origins and relatedness to classical and atypical BSE in other countries, an in-depth analysis of their PrP^res^ molecular and biochemical features was performed, including immunoreactivity, glycoform profiles, molecular size and relative proteinase K sensitivity of their PrP^res^ cores [2]. In addition, DNA sequencing was carried out on the full PrP-coding sequence in exon 3 of the *PRNP* gene in each of the 17 animals to assess the possibility of causative genetic mutations. The information obtained provides a better understanding of the epidemiology of BSE types in Canada and of how similar these types are to cases identified worldwide.

## Materials and Methods

### Animals and tissues

Sixteen of the seventeen Canadian BSE cases were detected by the Canadian BSE surveillance network and were confirmed by the National BSE Reference Laboratory. All positive cases were identified by active surveillance using immunohstochemistry (IHC) or rapid tests approved by the European Food Safety Authority (EFSA) and the Canadian Food Inspection Agency (CFIA). Confirmation was based on IHC and immunoblotting of scrapie-associated fibrils (SAF). Further analyses were done using a modified Hybrid Western Blot. One Canadian-born case was detected and confirmed using IHC by the National Veterinary Services Laboratory in Ames, Iowa, USA. The key details of each of the 17 Canadian BSE cases are summarized in Table 1.

**Table 1:** Animal information for the 17 Canadian BSE cases.

Case #	Date Confirmed	Cattle Breed	Age (yrs.)	Initial Typing
1	05/2003	Angus	5.8	C
2	01/2005	Holstein	8.2	C
3	01/2005	Charolais	6.8	C
4	01/2006	Holstein-Hereford	5.8	C
5	04/2006	Holstein	5.9	C
6	07/2006	Charolais X	∼16	H
7	07/2006	Jersey	4.2	C
8	08/2006	Charolais X	8–10	C
9	02/2007	Angus	6.6	C
10	05/2007	Holstein	5.5	C
11	12/2007	Hereford	13.8	L
12	02/2008	Holstein	6.1	C
13	06/2008	Holstein	5	C
14	08/2008	Gelbvieh X	6	C
15	11/2008	Holstein	7.8	C
16	05/2009	Holstein	6.7	C
US	12/2003	Holstein	6.7	C

### Tissue preparation and standard Prionics-Check Western blotting

Brain-stem tissue from each of the 17 cases was sampled at or close to the level of the obex. This tissue was used to prepare 10% (w/v) homogenates in 1 x Prionics homogenization buffer (Prionics-Check Western). Homogenization was performed for 45 s at 23,000 rpm in either a Prypcon container using the MediFASTH homogenizer (Consul AR) or in a Prioclip container using the Priogenizer homogenizer (Prionics AG). Initially, digestion of 100 µl of 10% homogenates was performed by adding 10 µl of digestion buffer (Prionics-Check Western) and 10 µl of PK (Prionics-Check Western), mixing thoroughly and incubating for 40 min at 48°C. The reactions were stopped by successive addition of 10 µl of digestion stop buffer (Prionics-Check Western), and 100 µl of 2× sample buffer (Prionics-Check Western). The samples were mixed well and then heated to 95°C for 5 min prior to gel electrophoresis and blotting.

Prior to analyses, the PrP^res^ amounts from each case were equalized by loading various volumes of the digested samples. Different amounts of disease-associated protein in each case was due to variables beyond our control including incubation time and the amount and frequency of the challenge dose. This step allowed for standardization of each sample input resulting in a better assessment of antibody immunoreactivity and clear protein band profiles for determining molecular weights and glycoform profiles. The loading amount which produced the strongest signal with three distinct bands was selected for the remaining experiments except where otherwise noted.

Each Canadian BSE case was also tested with the Prionics-Check Western blot using a number of modified protocols, as described in the following sections. The methods used for biochemical characterisation are even possible with autolysed samples which can pose a challenge for histological and immunohistochemical examination. Field cases are sometimes of poor tissue quality which can make strain typing based on morphologic observations very difficult.

### Proteinase K sensitivity

To determine the PK sensitivity of PrP^Sc^ in each of the Canadian BSE cases, phosphate- and Tris-based buffers (pH 6.5 and 8.0, respectively) designed for adjusting the tissue homogenate pH values were added [2]. After mixing 60 µl of 10% homogenate with an equal volume of pH-adjustment buffer, the desired pH of the sample was verified using 20 µl of the sample. Proteinase K (5 mg/ml) was then added to the pH-adjusted homogenates to reach a final concentration of 50 µg/ml (Ph 6.5) or 500 µg/ml (pH 8.0). Digestions were performed for 60 min at 37°C. The reaction was stopped by the successive addition of 10 µl of digestion stop buffer (Prionics-Check Western) and 100 µl of 2× sample buffer (Prionics-Check Western). The samples were mixed well and heated to 95°C for 5 min prior to gel electrophoresis and blotting.

### Deglycosylation with PNGase F

Deglycosylation of PrP^res^ using peptide *N*-glycosidase F (PNGase F; New England Biolabs, USA) was performed following the manufacturer's instructions with minor modifications. Briefly, PrP^res^ was denatured in a 3% (w/v) sodium dodecyl sulfate (SDS) solution instead of the denaturing buffer supplied. The treatment was carried out by incubating PK-digested, denatured PrP^res^ (5–10 µl of 10% homogenate) with PNGase F (final concentration 150 U/µl) for 16 hours at 37°C. The samples were mixed well with an equal volume of 2× sample buffer and heated to 95°C for 5 min before proceeding to gel electrophoresis and Western blotting.

### SDS-PAGE, Western blotting

The predetermined amount of each denatured sample (1.25–10 µl) was loaded on 12- or 17-well pre-cast 1 mm thickness, 12% Bis-Tris NuPAGE gels (Invitrogen, Canada). Electrophoresis was performed for 90 min at 100 V in 3-(N-morpholino)propanesulfonic acid (MOPS) running buffer (Invitrogen, Canada) with antioxidant (Invitrogen, Canada) using XCell SureLock Mini-Cells (Invitrogen, Canada). Magic Mark XP (Invitrogen, Canada) was used as a reference for molecular mass estimations. Electro-transfer, antibody binding, and chemiluminescence detection using CDP-Star and BioMax Light Chemiluminescence film (Kodak, United Kingdom) were performed following the Prionics-Check Western kit instructions (Prionics, Switzerland). Film images were then digitized to quantitate relative glycoform immunoreactivities using a BioRad ChemiDoc XRS (BioRad, Canada). The resulting image files were analyzed using the BioRad Quantity One software version 4.6.3 (BioRad, Canada), and the integrated optical densities and apparent molecular masses of the bands were exported for further calculations. Only bands with molecular masses ranging from 30 to 16 kDa were used to calculate the glycoform profile, in which the total integrated optical density of each glycoform band was expressed as a percentage of the summed integrated optical densities of the three bands in the triplet.

### Antibodies and epitope-specific antibody reactivity

To compare epitope-specific PrP^res^ antibody reactivities, the Prionics-Check Western kit specific monoclonal antibody (mAB) 6H4 was replaced in turn by each member of a panel of selected antibodies. Antibodies 12B2, P4 and SAF32 are PrP N-terminal-specific antibodies, antibodies 9A2, 6H4 and L42 are PrP core-specific antibodies and antibodies 94B4 and SAF84 are the PrP C-terminal-specific antibodies (Table 2).

**Table 2:** Antibodies used to determine differential immunoreactivity.

Antibody	Eliciting Peptide	Target Epitope	Epitope reference	Source/Working Conc.
**12B2**	bovine PrP 97-115	101WGQGG105	[33]	J. Langeveld, CVI/0.5 µg/ml
**P4**	ovine PrP 89-104	85WGQGGSH93	[34]	Rida/Germany/0.2 µg/ml
**SAF 32**	ovine PrP59-89	62/70/78/86QPHGGGW68/76/84/92	[35]	SPI-BIO, France/0.1 µg/ml
**9A2**	bovine PrP 97-115	110WNK112	[33]	J. Langeveld, CVI/0.5 µg/ml
**6H4**	ovine PrP148-157	156DYEDRYYREN164	[36]	Prionics AG/Kit dilution
**L42**	ovinePrP145-163	156YEDRYY161	[37]	Rida, Germany/0.02 µg/ml
**94B4**	ovine PrP190-197	198HTVTTTTK205	[2]	J. Langeveld, CVI/0.2 µg/ml
**SAF84**	bovine PrP145-169	175RPVDQY180	[35]	J. Grassi, CEA/1.0 µg/ml

The antibodies used can be organized into three groups based on their target epitope. The N-terminal-specific antibodies include 12B2, P4, and SAF32, core-specific antibodies are 9A2, 6H4, and L42, and the C-terminal-specific antibodies are 94B4 and SAF84.

### DNA extraction and sequencing

Genomic DNA was extracted from 0.2–0.4 g unfixed brain tissue on different days to avoid cross-contamination, using a standard method (Proteinase K/phenol-chloroform/ethanol precipitation). PCR primers BTAPRNPDS9:19471U22 and BTAPRNPDS9:21426L20 were used with published PCR conditions and sequencing primers [28] to amplify and sequence the entire PrP-coding sequence located in exon 3 of the bovine *PRNP* gene from all 17 BSE-affected cattle, using BigDye® dideoxy cycle-sequencing chemistry (Applied Biosystems, USA). Sequence contigs were assembled, visually inspected and analyzed with LaserGene™ (DNAStar, USA) software.

## Results

In this study, we analyzed the molecular features of PrP^res^ using both a standard and modified Prionics-Check Western Blot techniques. The characteristics used for typing the Canadian BSE cases are based on the molecular characterization of natural BSE case from other countries. Using these characteristics, the initial confirmatory classifications were supported resulting in 15 C-, 1 H- and 1 L-type Canadian BSE cases.

### Molecular weights and glycoform profiles of PrP^res^

As part of the initial confirmation, all Canadian BSE cases were classified as C-, H-, or L-type on the basis of PrP^res^ molecular weights and relative antibody immunoreactivities. In the 15 Canadian C-type BSE cases, the mean (± SD) relative immunoreactivities of di-, mono-, and unglycosylated moieties were 62.5±5.8, 25.6±2.5 and 12.0±4.5%, respectively. The molecular-mass estimates were 27.5±0.5, 21.8±0.7 and 17.6±0.6 kDa for the di-, mono- and unglycosylated bands, respectively. In the L-type case, the relative immunoreactivities were 39.3±0.6, 35.0±1.0 and 26.3±1.2%, and estimated molecular masses of the di-, mono- and unglycosylated moieties were all slightly lower (by ∼1 kDa) than those of the C-type, at 26.3±0.6, 20.7±0.6 and 17.3±0.6 kDa, respectively. The single H-type case had higher molecular masses (by ∼1.5 kDa) for all three bands, with di-, mono- and unglycosylated forms at 28.7±1.5, 23.3±1.2 and 19.3±1.2 kDa, respectively. When using core-specific or N-terminal-specific antibodies, the glycoform profile of the Canadian H-type case resembled the C-type cases with 58.3±2.5% digycosylated, 29.3±0.6% monoglycosylated, and 12.7±3.5% unglycosylated PrP^res^. When using a C-terminal antibody, however, the molecular weight (27.7±0.6, 22.7±0.6 and 17.7±0.6 kDa) was more similar to that of the C-type, and the glycoprofile (38.3±0.6, 36.7±0.6 and 25.0±1.0%) was more similar to the L-type BSE cases.

To directly compare the PrP^res^ glycoform ratios from each case, the mean proportion of diglycosylated glycoform immunoreactivity was divided by the mean proportion of monoglycosylated glycoform immunoreactivity [16], [29]. In the Canadian L-type case, this ratio was 1.1. When this ratio was determined for the C- type cases, the values ranged from 2.1 to 3.7. The H-type ratio was similar to that of the C-types when using a core-specific antibody with a value of 2.0, however when using a C-terminal antibody this ratio shifted down to a value of 1.2 (Table 3).

**Table 3:** Glycoform quantification of PrP^res^ from the Canadian BSE cases using core antibody 6H4.

*CASE/Type*	*D*	*M*	*Un*	*D/M*	*CASE/Type*	*D*	*M*	*Un*	*D/M*
**1(C)**	60.9	27.1	12.0	**2.2**	**9(C)**	75.6	20.4	4.2	**3.7**
**2(C)**	61.0	26.7	12.2	**2.3**	**10(C)**	61.1	24.8	14.1	**2.5**
**3(C)**	56.2	27.0	16.9	**2.1**	**11(L)**	39.3	35.0	26.3	**1.1**
**4(C)**	58.3	26.7	15.0	**2.2**	**12(C)**	60.7	27.4	11.9	**2.2**
**5(C)**	58.1	24.7	17.2	**2.4**	**13(C)**	58.4	25.8	15.8	**2.3**
**6(H)**	58.3	29.0	12.7	**2.0**	**14(C)**	61.7	23.3	15.1	**2.6**
**7(C)**	75.2	20.3	4.5	**3.7**	**15(C)**	63.9	28.8	7.1	**2.2**
**8(C)**	58.6	25.6	15.8	**2.3**	**16 (C)**	61.0	26.8	12.2	**2.3**
**US case (C)**	66.5	28.2	5.3	**2.4**					

The proportion of PrP^res^ detected as diglycosylated is approximately 60% for the C- and H-type. In comparison, the L-type had a much lower proportion of the diglycosylated PrP^res^ at approximately 40%. The L-type case also had a ratio of di- to monoglycosylated (D/M) PrP^res^ well below 2 (1.1), whereas the ratios of intensity of D/M from all other cases were above or equal to 2 when using core specific antibodies for detection. Profiles were determined by analyzing western blot reactivity using the BioRad Quantity One software (BioRad, Canada).

***D***  =  Diglycosylated PrP^res^; ***M***  =  Monoglycosylated PrP^res^; ***Un***  =  Unglycosylated PrP^res^; ***D/M***  =  Diglycosylated PrP^res^/Monoglycosylated PrP^res^.

### Deglycosylation

Variations in the molecular weights of the PrP^res^ core associated with atypical BSE cases are often difficult to determine when all glycoforms are present, especially in L-type BSE which often displays a subtle band shift. Removal of the carbohydrate moieties from PrP^res^ offers a way to enhance the estimates of the molecular-weight shifts in the L- and H-type PrP^res^ cores.

**Figure 1:**
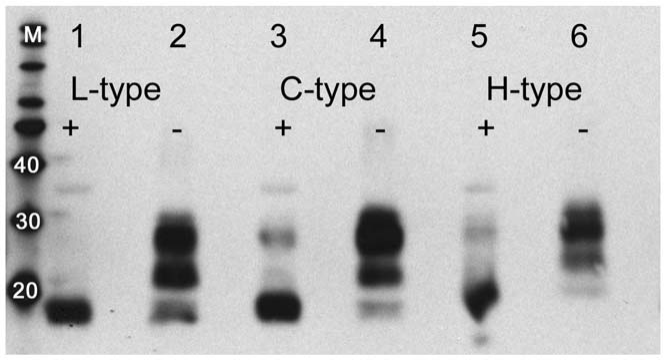
Western blot analysis of Canadian BSE after PK digestion and PNGase F treatment. L- (case 11, lanes 1 and 2), C- (case 12, lanes 3 and 4) and H- (case 6, lanes 5 and 6) type BSE after PK digestions with (+) and without (−) PNGase F deglycosylation. Core antibody 6H4 detection demonstrates the size differences between classical and atypical BSE and also a slight size shift between the unglycosylated and deglycosylated bands within each individual type of BSE. The lane labelled as M contains the molecular weight marker and weights are in kilodaltons.

For each of the BSE types, PNGase F treatment resulted in a subtle change in electrophoretic migration of the deglycosylated band when compared to the naturally occurring unglycosylated isoform (Fig. 1). None of the PNGase-treated samples showed complete removal of all carbohydrates, and the susceptibility to this treatment appeared type-dependent with the L-type Canadian BSE being the most completely deglycosylated. Deglycosylation of the H-type PrP^res^ also resulted in the presence of two unglycosylated protein isoforms when using C-terminal antibodies for detection, one at 19 kDa and the other at 10 kDa (data not shown).

### Resistance to PK digestion

**Figure 2:**
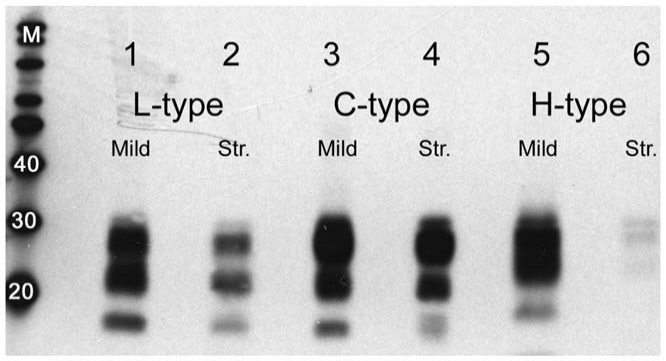
Western blot analysis of Canadian BSE after mild and stringent PK digestion. L- (case 11, lanes 1 and 2), C- (case 12, lanes 3 and 4) and H- (case 6, lanes 5 and 6) type BSE after PK digestions at 37°C for one hour under mild (Mild: pH 6.5, PK 50 µg/ml) and stringent (Str.: pH 8.0, PK 500 µg/ml) conditions. Core antibody 6H4 detection demonstrates little change in the amount of PrP^res^ surviving the stringent digestion in C-type BSE and a significant degradation of L-type PrP^res^ and almost complete degradation of H-type PrP^res^ under the stringent conditions. The lane labelled as M contains the molecular weight marker and weights are in kilodaltons.

Proteinase K (PK) digestion under mild and stringent conditions revealed that the misfolded prion protein from C-type BSE is more PK-resistant than that from H- or L-type atypical BSE. Under mild conditions, the full-length PrP^C^ was completely degraded, while PrP^Sc^ was converted to readily visualized amounts of PrP^res^ for all of the BSE cases. Under stringent conditions in contrast, the remaining film signals of L- and H-type PrP^res^ decreased significantly, while that of the C-type PrP^res^ remained nearly at the same level. Densitometric quantification revealed that the C-type BSE PrP^res^ only decreased by approximately 10% under the stringent conditions while the decrease in surviving PrP^res^ was greater than 50% for both L- and H-type cases (Fig. 2).

### Investigation of PrP^res^ immunoreactivities using different antibodies

When N-terminal-specific, C-terminal-specific and core-specific antibodies were compared for their relative abilities to detect PrP^res^ in the different types of BSE, further evidence supporting the initial classification of the different BSE types was established. The N-terminal-specific antibodies 12B2, P4 and SAF32 bound to all three bands of PrP^res^ in H-type BSE, but very weakly or not at all to the PrP^res^ bands of the C- or L-type BSE. The core-specific and C-terminal-specific antibodies 9A2, L42, 6H4, 94B4 and SAF84 bound to all three bands of the PrP^res^ of the H-, L-, and C-type BSE (Table 4). Both C-terminal-specific antibodies also bound to a fourth band at around 10 kDa in the H-type BSE case.

**Table 4:** Western blot PrP^res^ immunoreactivity profiles for the Canadian BSE cases.

Ab	1C	2C	3C	4C	5C	6H	7C	8C	9C	10C	11L	12C	13C	14C	15C	16C	US C
12B2	*	*	*	*	*	***	—	*	—	*	*	*	*	*	—	—	—
P4	—	—	—	—	—	**	—	—	—	—	—	—	—	—	—	—	—
SAF32	—	—	—	—	—	**	—	—	—	—	—	—	—	—	—	—	—
9A2	***	***	***	***	***	***	**	***	**	***	***	***	***	***	***	***	***
6H4	****	****	****	****	****	****	***	****	**	****	****	****	****	****	****	****	****
L42	***	***	***	***	***	***	**	***	*	***	***	***	***	***	***	***	***
94B4	***	***	***	***	***	***	**	***	*	***	***	***	***	***	***	***	***
SAF84	***	***	***	***	***	***	**	***	*	***	***	***	***	***	***	***	***

When using N-terminal-specific, C-terminal-specific and core-specific antibodies to compare their affinity to the different types of BSE, N-terminal-specific antibodies P4, 12B2 and SAF32 bound to all three bands of PrP^res^ in H-type, but not to the PrP^res^ bands of the C- or L-type cases. The core- and C-terminal-specific antibodies 9A2, L42, 6H4, 94B4, SAF84 bound to all three bands of the PrP^res^ of the H-, L-, and C-types.

****All three bands of PrP^res^ were visible and strongly reactive;

***All three bands of PrP^res^ were visible;

**Two of the three bands of PrP^res^ were visible;

*One of the three bands of PrP^res^ was visible; - None of the PrP^res^ bands were visible; Ab  =  Primary antibody.

### DNA sequencing

A summary of diploid genotypes of the PrP-coding regions in *PRNP* exon 3 is provided in Table 5 for each of the 17 BSE cases in relation to a reference sequence (GenBank accession AJ298878). Although 4 previously known polymorphisms were observed, all of these were either translationally silent (CAG↔CAA in codon 78, CCC↔CCT in codon 113 and AAC↔AAT in codon 192) or have been shown previously to have no phenotypic association with BSE (OPR region carrying either 5 or 6 tandem repeat units).

**Table 5:** Genotypes of Canadian BSE-affected cattle at polymorphic positions in the protein-coding sequence of *PRNP*.

	PrP Codon(s)			
Sequence	OPR (54–103)	78 (Gln)	113 (Pro)	192 (Asn)
**AJ298878**	6	CAG	CCC	AAC
**Case 1**	..	..	CCY	..
**Case 2**	..	CAA	..	..
**Case 3**	..	..	..	..
**Case 4**	..	CAR	..	..
**Case 5**	..	CAA	..	..
**Case 6**	..	..	..	..
**Case 7**	..	..	..	..
**Case 8**	..	..	..	AAY
**Case 9**	..	CAR	..	..
**Case 10**	..	..	..	..
**Case 11**	..	CAR	..	AAY
**Case 12**	..	..	..	..
**Case 13**	..	CAR	..	..
**Case 14**	..	CAR	..	..
**Case 15**	..	..	..	..
**Case 16**	6/5	CAG/–	..	..
**US Case**	..	..	..	AAY

AJ298878  =  GenBank reference sequence; OPR  =  octapeptide repeat; ..  =  homozygous for AJ298878 sequence; –  =  codon 78 missing on 5-OPR allele.

## Discussion

The classification of BSE types based on molecular and biochemical properties involves molecular weight determination of PrP^res^. Atypical BSEs have a higher or lower apparent molecular mass of PrP^res^ when compared to C-type BSE using WB analysis with core-specific antibodies. Such differences are in the range of 0.9 to 1.3 kDa for the H-type, but rather subtle for L-type (∼0.3 kDa) [2], [3], [7], [10]. The Canadian H-type BSE case, detected in 2006, displays an obvious molecular weight shift of around 1.3 kDa, similar to that observed in some cases in other countries [2], [3]. A lower molecular mass of PrP^res^ for L-type BSE compared with C-type BSE has been recorded in cases from Italy, France, Japan, German, Poland and the Netherlands [2], [3], [13], [15], [16], [20], [29]. The Canadian L-type BSE case has a subtle molecular weight shift downward compared to Canadian C-type cases, which made it initially challenging to confirm and additional supporting evidence was necessary.

PNGase F treatment to remove associated carbohydrates from glycosylated PrP^res^, which is intended to generate a single unglycosylated protein species for more precise molecular-weight estimation, provided further support for the size shifts in Canadian H- and L-type BSE cases detected in the initial analyses. Removal of carbohydrates also confirmed the presence of two sizes of unglycosylated PK-resistant fragments, at approximately 19 and 10 kDa, in the H-type case. Similar behaviour has been previously observed and was explained by the possibility of two subpopulations of PrP^Sc^ carrying two available PK-cleavage sites with different susceptibilities to digestion in H-type cases [2]. PNGase F treatment results in some residual glycosylated PrP^res^, even under the most highly denaturing conditions. Susceptibility to deglycosylation was noted to be associated with BSE type, with PNGase F treatment being most effective on the L-type case and less effective on the H- and C-type Canadian cases. Presuming that glycosylation occurs on the same amino acid residues in all cases, this may be related to differences in the conformation and/or conformational stability of the different BSE types.

Another interesting observation in the deglycosylation experiments was the slightly lower migration rate of the deglycosylated band of each BSE type after treatment with PNGase F compared to the rate for the unglycosylated band in untreated samples (Fig. 1). This is believed to result from the N-linked carbohydrate removal process, which alters asparagine to aspartic acid and thus reduces electrophoretic mobility of protein-detergent complexes due to a decrease in the SDS:PrP^res^ molar ratio [2].

While removing carbohydrate groups is useful for molecular weight analysis, retaining these post-translational modifications is also valuable for BSE strain typing. In general, L-type BSE cases have a unique glycoform profile which includes a smaller proportion of diglycosylated PrP^res^ and a larger proportion of mono- and unglycosylated PrP^res^ when compared to C- and H-type [2], [3], [13], [16], [20], [29]. This more balanced di- to monoglycosylated ratio is also seen in the Canadian L-type BSE case. In Canadian C-type and H-type cases, the average ratio of di- to monoglycosylated PrP^res^ is consistently ≥2, while in the L-type case it is <1.5. When using C-terminal antibodies however, the Canadian H-type BSE di- to monoglycosylated ratio shifted closer to the L-type ratio. It was recently suggested that this may reflect the presence of a second population of PrP^res^ in H-type BSE with a slightly lower molecular weight [30]. This would result in the second population's diglycosylated band merging with the monoglycosylated band of the first population increasing its apparent abundance. Based on the fact that the altered glycoprofile and fourth band are only seen with C-terminal antibodies [30], it seems that this second PrP^res^ population is cleaved between the epitopes detected by the core-specific and C-terminal-specific antibodies which could result in the observed ∼10 kDa size shift.

Conformational stability can also vary in different prion disease strains. This can be analyzed in different ways, but one of the simplest is to determine the proteinase K sensitivity of the misfolded protein using mild and stringent conditions [2]. The H- and L-type BSE isolates displayed higher susceptibilities to PK digestion than the C-type isolates, suggesting that the conformation of the Canadian C-type BSE is more stable than the two atypical forms. This is similar to results from comparable analyses on C-type and atypical BSE done elsewhere [2], [21].

Proteinase K digestion under mild conditions induces N-terminal cleavage of PrP^Sc^ in all three BSE types to varying degrees. Selecting antibodies with epitopes localized on either side of the cleavage sites allows for another mechanism of BSE type differentiation. It has been demonstrated that C- and L-type BSE react only weakly with antibodies directed towards the N-terminal portion of PrP^res^ (e.g., 12B2 antibody [2], [16]). This was also shown for Canadian cases. When testing Canadian H-type BSE, as is the case with other H-type BSE cases, there is strong binding with the N-terminal antibodies including 12B2, P4 and SAF32. Another similarity between Canadian H-type BSE and those found in other countries is the detection of a fourth band at approximately 10 kDa when using C-terminal-specific antibodies. This unique behaviour of H-type BSE needs further investigation to determine its source and what implications this may have on other H-type characteristics such as pathogenetic traits.

In May 2003, after the detection of the first indigenous case of BSE, Canadian surveillance was modified to increase test numbers to demonstrate the extremely low level of BSE in the country. This additional effort primarily targets “4D” cattle over 30 months of age. This includes animals that are dead, non-ambulatory (down), sick at *ante mortem* inspection (diseased), or presented for emergency slaughter (distressed). The program also includes animals of any age presenting clinical signs consistent with BSE. As of November 2009, this program had tested over 275,000 cattle as negative and 16 cattle as positive for BSE. An additional case was detected in the United States and traced back to Canada, for a total of 17 Canadian-born cases. The aim of our study was to provide an in-depth analysis of the molecular and biochemical properties of the PrP^res^ associated with each of these cases and thus insight into the epidemiology of BSE in Canada. We confirmed the diagnoses of 15 C-, 1 L- and 1 H-type BSE cases by determining molecular mass, immunoreactivity, proteolytic sensitivity (stability) and glycoform profile.

In addition to shared molecular characteristics, a common feature of the majority of atypical BSE cases worldwide is their occurrence in older animals as compared to C-type BSE which is found in various age groups [16]. Most atypical BSE cases have occurred in animals over 8 years old with an average age of 12 years [2], [3], [10], [13], [15], [16], [29]. The Canadian atypical BSE cases occurred in a 16-year-old Charolais (H-type) and a 13.8-year-old Polled Hereford (L-type), both with clinical disease at the time of sampling. French, Italian, Dutch, Polish, and German atypical BSE cases have occurred in healthy slaughter animals but have also been reported among fallen stock, and these might have displayed unreported clinical abnormalities [2]. This variation in clinical presentation adds to the list of questions that remain unanswered for these types of BSE.

The origin of atypical BSE is unknown, but sporadic, infectious and genetic mechanisms have all been suggested. Several groups have argued that these cases may represent the existence of a sporadic prion disease in bovines, perhaps similar in etiology to sporadic Creutzfeldt-Jakob disease in humans [2], [6], [12], [19]. Experimental infection of cattle with an isolate of naturally occurring British sheep scrapie resulted in differences in the PrP^res^ electrophoretic profiles compared to classical C-type BSE which led to the suggestion that atypical BSE may be a result of transmission of a prion disease from a different natural host into cattle [14]. In addition, an American H-type BSE case with a mutation (E211K) in the *PRNP* gene has been reported [19]. This represents the first case of BSE with a potentially pathogenic mutation within the bovine *PRNP* gene, and experiments are underway to determine the potential importance of this mutation in the development of prion disease in cattle [19]. We did not observe such mutations in the Canadian BSE cases analyzed as of November 2009, and negative results of a large population survey in US cattle strongly suggest that the E211K allele is not common in North American cattle [31]. However, the intrinsically recurrent nature of genetic mutation, as shown particularly for the homologous E200K mutation known to cause genetic Creutzfeldt-Jakob disease in humans [32], means that the possibility that a small subpopulation of cattle that carry such mutations exist cannot be eliminated.

Our results indicate that the range of molecular characteristics of misfolded PrP in Canadian BSE cases is very similar to that observed in other countries and suggests a number of criteria to use when typing BSE cases (Table 6). It is also interesting that the Canadian atypical BSE cases match so well with cases from other countries in terms of their epidemiological profiles, including detection in older animals and no definitive cause of disease. Ongoing inoculation studies with Canadian BSE types in cattle and transgenic mice will provide clarification on how similar these isolates are after transmission using defined and controlled experimental inoculations. These studies should help to answer some of the many outstanding questions about atypical BSE and aid in policy development to reduce the risk of atypical BSE transmission to animals and humans.

**Table 6:** Summary of the molecular/biochemical properties of PrP^res^ used to discriminate between the three BSE types.

*Case #*	*Core Ab Reactivity Glycoprofile Ratio (D/M)*	*C-term. Ab Reactivity Glycoprofile Ratio (D/M)*	*N-term. Ab Reactivity*	*Proteolytic susceptibility (S/M)*	* Size determination of PrP^res^ core *	*BSE type*
**1–5, 7–10,**	>2	>2	No	Most stable	Reference	C
**12–16, US**				∼1	∼29 kDa digly.	
**6**	>2	<2	Yes	Most sensitive	Higher	H
				≪1	∼2 kDa	
**11**	<2	<2	No	Sensitive	Lower	L
				<1	∼1 kDa	

Determination of these five characteristics provides valuable information in typing BSE cases. Each of the Canadian cases were definitively place it into one of the three know types of BSE after reviewing the results generated when determining these five characteristics.

**Ab  = ** Antibody, **D/M**  =  Diglycosylated PrP^res^/Monoglycosylated PrP^res^, **S/M**  =  stringent digest immunoreactivity/mild digest immunoreactivity.
